# Effect of head-down tilt on clinical outcome and cerebral perfusion in ischemic stroke patients: A case series

**DOI:** 10.3389/fneur.2022.992885

**Published:** 2022-09-26

**Authors:** Zi-Ai Zhao, Nan-Nan Zhang, Lin Tao, Yu Cui, Meng Li, Shou-Liang Qi, Hui-Sheng Chen

**Affiliations:** ^1^Department of Neurology, General Hospital of Northern Theater Command, Shenyang, China; ^2^School of Sino-Dutch Biomedical and Information Engineering, Northeastern University, Shenyang, China

**Keywords:** arterial spin labeling, cerebral blood flow, head-down tilt, stroke, early neurological deterioration

## Abstract

**Background:**

The effect of head position on stroke is not clear. The current study aimed to observe the effect of head-down tilt on acute ischemic stroke (AIS) patients with large vessel occlusion.

**Methods:**

We observed the influence of head-down tilt position on clinical outcomes, myocardial enzymogram and N-terminal pro b-type Natriuretic Peptide in 4 AIS patients who suffered early neurological deterioration (END). Cerebral perfusion imaging was performed in 3 patients using arterial spin labeling.

**Results:**

In series of AIS patients with END, head down tilt (-20°) prevented further neurological deterioration and improved clinical outcomes. An increase in cerebral blood flow was observed by arterial spin labeling after head down tilt treatment. No obvious adverse events occurred.

**Conclusion:**

The case series suggest that head-down tilt may improve clinical outcome in AIS patients through increasing the cerebral perfusion with no obvious adverse events. The finding needs to be confirmed in future clinical trials.

## Introduction

The prognosis in patients with acute ischemic stroke (AIS) depend on the location and size of the occluded cerebral vessel (1), the extent of collateral blood flow (2, 3) and the time to reperfusion therapy (4). Head position after ischemic stroke affects cerebral blood flow (CBF), intracranial pressure, blood flow velocity, cerebral perfusion pressure, oxygenation, as well as other factors which are closely related to the prognosis after ischemic stroke (5, 6). Promoting blood flow during the acute phase of ischemic stroke may directly influence the development of brain infarct and clinical deficit (7). Until now, for the acute ischemic stroke patients, the question of optimal body position has not been fully addressed (4).

The previous studies about the influence of lying-flat vs. sitting-up position on clinical outcome after stroke were controversial. Comparing with an upright position, lying flat induces a significant increase in intracranial pressure in patients with brain injury (8, 9). Moderate head elevation is a standard practice in the management of intracranial pressure (10). Conversely, in AIS patients, some studies indicated that an increase in blood flow velocity and cerebral perfusion pressure could be achieved after changing the upright position to lying flat (11–14), which will promote residual blood flow to ischemic brain tissue (15). However, the HeadPoST (Head Position in Acute Stroke Trial) study did not find the difference in disability outcomes between AIS patients assigned to a lying-flat position and sitting-up position with the head elevated to at least 30° for 24 h (6). We contend that the aggressive head position such as head-down tilt can exert neuroprotective effect in particular AIS patients such as those with large vessel occlusion (LVO). To the best of knowledge, there was no study to investigate the effect of head down tilt position on neurological outcome of ischemic stroke patients.

Head-down tilt may improve clinical outcome of AIS patients through increasing collateral blood flow and CBF. CBF refers to perfusion per unit of tissue, and is optimally measured with a diffusible tracer that can exchange between the blood and the brain. In arterial spin labeling (ASL), the diffusible tracer is a magnetic label applied to blood water molecules, produced by saturating or inverting the longitudinal component of the magnetic resonance signal (16). ASL perfusion magnetic resonance imaging (MRI) sequences are increasingly being used to provide non-invasive MR-based CBF quantification. Clinically, ASL has mainly been used in cerebrovascular disease including stroke (17), steno-occlusive disease (18), arteriovenous malformation (19), and Moyamoya disease (20). Comparing with transcranial Doppler, which only provided indirect information of CBF, ASL perfusion imaging provides quantification of CBF. Additionally, ASL can be performed routinely and repeatedly without contrast administration or ionizing radiation (21).

In this study, we observed the influence of head down position on clinical outcomes and CBF in a small group of AIS patients who suffered neurological deterioration.

## Methods

### Participants

**Figure 1:**
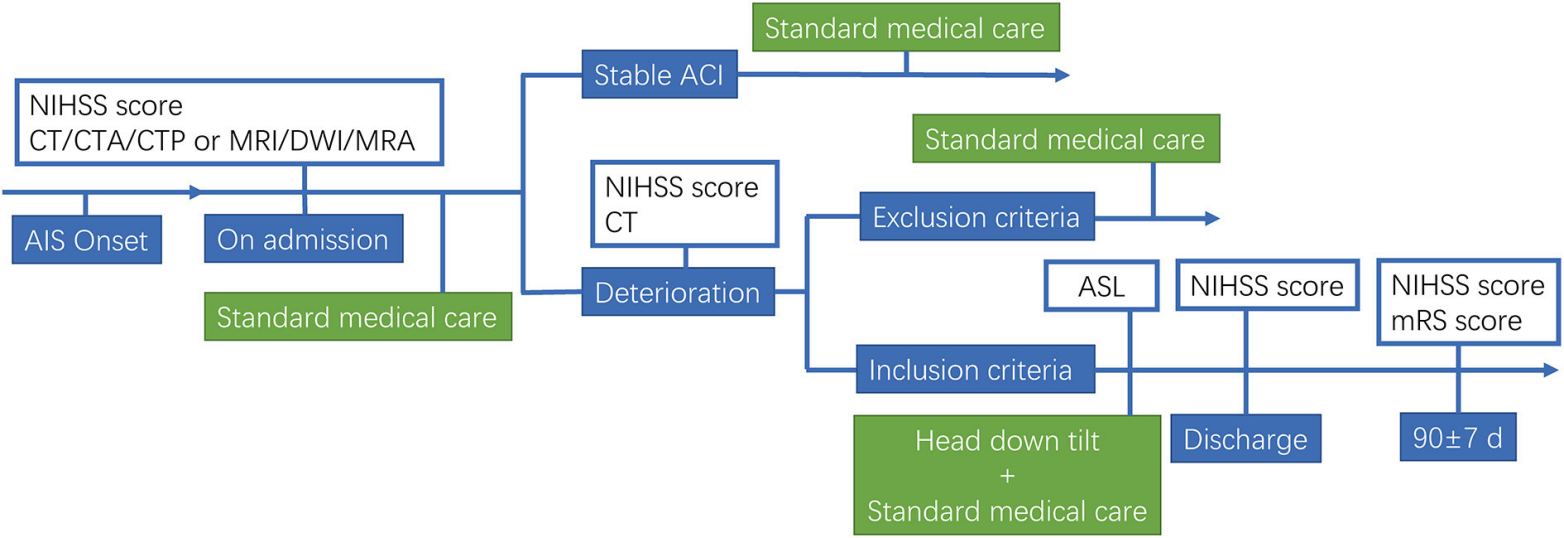
Flow diagram of the study.

We performed a pilot trial in AIS patients. This study was approved by the Ethics Committee of former General Hospital of Shenyang Military Region (No. k2017-10). Informed consent was obtained from all study subjects, and the study was performed in accordance with the ethical standards as laid down in the 1964 Declaration of Helsinki and its later amendments or comparable ethical standards. Main inclusion Criteria: (1) age ≥18 years; (2) AIS with large vessel occlusion; (3) the patients occurred neurological deterioration within 7 days of onset; (4) 6–16 NIHSS after deterioration; (5) first stroke onset or past stroke without neurological deficit (modified Rankin Scale, mRS = 0); (6) fully understand and cooperate with the doctor's instructions; (7) the availability of informed consent. Exclusion Criteria: (1) Hemorrhagic stroke or mixed stroke; (2) combined with severe organ dysfunction; (3) a history of hemorrhagic stroke; (4) a history of stroke with severe sequelae; (5) planning revascularization in 3 months; (6) ischemic stroke due to surgical intervention; (7) participating in other clinical trials within 3 months; (8) pregnant or lactating women. Early neurological deterioration (END) was defined as an increase of 2 points or more on the NIHSS, except for cerebral hemorrhage. Schematic for head down tilt treatment of AIS patients is shown in Figure 1.

### Head down tilt treatment in AIS patients

After the informed consent was signed, the patient was treated with head down tilt as follows. The patients lay supine on tilt platform bed, which was adjusted to −20° for about 60 min for one time. During the treatment, a 3–5 min break to lay flat was allowed for patients who cannot tolerate head down tilt position. Head down tilt treatment was performed 3 times daily for 7 days. During the treatments, the patients were continuously monitored by ECG and blood oxygen saturation, and the side-lying head down position with −20° was allowed if high risk of aspiration was suspected.

### Arterial spin labeling

After finishing the first treatment of head down tilt, ASL was performed to determine the influence of head down tilt on cerebral blood flow. In the ASL examination, nodes can be ideally described as regions which have coherent patterns of connections in anatomical structure or function in brain. Different parcellation methods of brain regions caused distinct brain network constructions, usually the entire brain surface would be covered completely by the parcellation method. The procedure to determine nodes in the study is as follows. First, the PdWI image of every subject was linearly registered to T1-weighted image and the result matrix was obtained. The T1-weighted image was normalized into MNI space, using both linear registration and non-linear registration. The transformation field derived from T1 to MNI normalization warped the co-registered PdWI image in structural space. Both of the warp-fields and transformation matrix were inverted by the command of convert_xfm and invwarp respectively, and then they were applied to the Human Brainnetome Atlas which is based on connectional architecture. At last, the atlas registered to each subject was resampled to CBF pcolor image, then 246 regions were obtained by using this procedure and each region represents a node in the brain network. Formula for the calculation is as follow.


$$\begin{array}{l} CBF=\frac{6000\;\cdot \lambda \;\cdot \left(SI_{control}-SI_{label}\right)\;\cdot e^{\frac{PLD}{T_{1,blood}}}}{2\;\cdot \alpha \;\cdot T_{1,blood}\;\cdot SI_{PD}\;\cdot \left(1-e^{-\frac{\tau }{T_{1,blood}}}\right)} \end{array}$$

### Statistical analysis

Results were presented as mean values ± standard deviation (SD). The comparisons of the changes in myocardial enzymogram and NT-proBNP before and after head down tilt position were analyzed by Paired t- test. A value of *p* < 0.05 was considered statistically significant.

### Data availability

The data that support the findings of this study are available from the corresponding author upon reasonable request.

## Results

### Head down tilt position alleviates clinical outcomes in acute ischemic stroke patients with LVO

In the present study, 4 eligible AIS patients who occurred END within 7 days after onset were treated with head down tilt position (ages 48–74, average 60 years). Baseline characteristics of patients, symptoms, culprit arteries, etiology, presence of cervical artery atherosclerosis, onset to head down tilt time, prior intravenous thrombosis, prior mechanical thrombectomy, NIHSS score at different stages, and adverse events were listed in Table 1. The results showed that neurological function did not further deteriorate after head down tilt, and neurological deficit at discharge and neurological outcome 90 days after onset were significantly improved. No patients reported obvious uncomfortable events. The electrocardiographic monitoring did not show visible change during head down tilt. There were no changes in myocardial enzymogram and N-terminal pro b-type Natriuretic Peptide (NT-proBNP) before and after head down tilt in all patients (Table 2).

**Table 1:** Characteristics of patients.

	**Case 1**	**Case 2**	**Case 3**	**Case 4**
Age	56	74	62	48
Symptoms	Limb weakness, limb ataxia, dysarthria and facial palsy	Limb weakness, dysarthria and sensory loss	Drowsy, dysarthria, limb weakness, facial palsy, partial hemianopia	Severe aphasia, Limb weakness
Culprit arteries	Middle cerebral artery	Basilar artery	Vertebral artery	Middle cerebral artery
Etiology	Atherosclerosis	Atherosclerosis	Atherosclerosis	Atherosclerosis
Presence of cervical artery atherosclerosis (carotid or vertebral/basilar artery)	Yes	Yes	Yes	Yes
Intravenous thrombolysis	No	No	No	No
Mechanical thrombectomy	No	No	No	No
Baseline NIHSS score	6	6	6	2
NIHSS score at END (Onset to END time)	8 (22.3 h)	9 (24.5 h)	8 (122.1 h)	6 (48.6 h)
Onset to HDT time	22.65 h	24.9 h	122.65 h	49.3 h
NIHSS score at discharge (Onset to discharge time)	3 (12 d)	5 (10 d)	6 (11 d)	4 (12d)
mRS at 90 days	1	1	2	1

NIHSS, National Institute of Health stroke scale; END, early neurological deterioration; HDT, head down tilt; mRS, modified Rankin Scale.

**Table 2:** Changes in myocardial enzymogram and NT-proBNP before and after head down tilt position.

	**CK (U/L)**		**CKMB (U/L)**		**LDH (U/L)**		**NT-ProBNP (pg/mL)**	
	**Before**	**After**	**Before**	**After**	**Before**	**After**	**Before**	**After**
Case 1	65	63	10	15	197	184	20.55	19.03
Case 2	75	70	12	14	207	204	50.92	90.08
Case 3	129	136	15	13	222	236	1,840.31	1,732.67
Case 4	106	107	18	21	172	164	31.22	33.04
Mean ± SD	93.75 ± 29.27	94.00 ± 34.01	13.75 ± 3.50	15.75 ± 3.59	199.50 ± 21.01	197.00 ± 30.70	485.75 ± 903.12	468.71 ± 843.20
Paired *t*-test	t = −0.0976 *p* = 0.928		t = −1.359 *p* = 0.267		t = 0.426 *p*= 0.699		t = 0.540 *p* = 0.627	

CK, creatine kinase; LDH, lactate dehydrogenase; NT-ProBNP, N-terminal pro-B type natriuretic peptide.

### Head down tilt increases cerebral blood flow in patients with AIS

**Figure 2:**
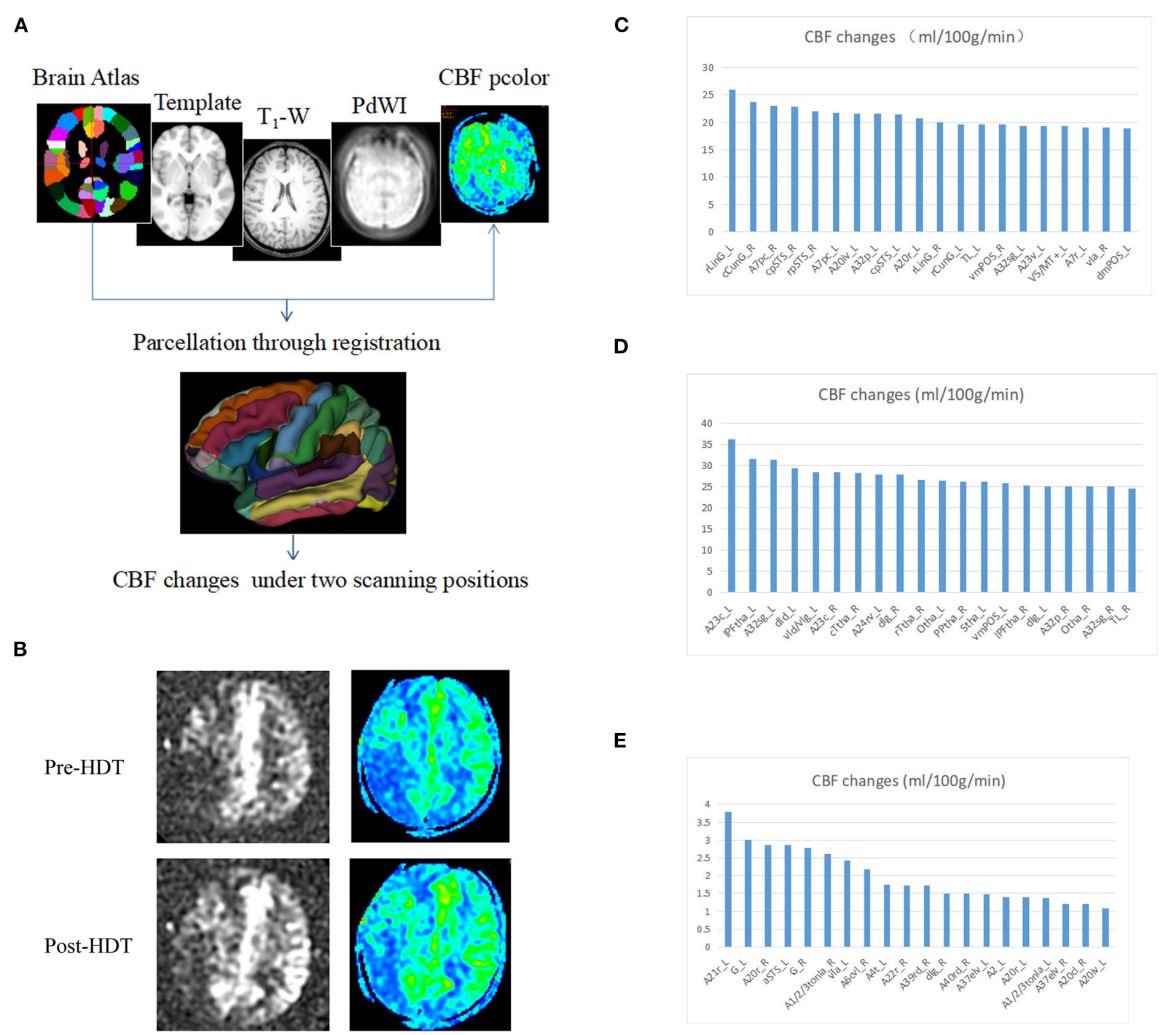
Changes in cerebral blood flow after head down tilt position. **(A)** Pipeline for the registration and determination of regions. The Brainnetome Atlas, template, T1-weighted, PdWI and CBF pcolor were used to co-registration and 246 regions of interest were obtained in every subject. In addition, the CBF changes were calculated under two scanning positions (head down tilt position and lying flat position). **(B)** Representative ASL and CBF pcolor images from Case 1. **(C)** CBF changes in Case 1. **(D)** CBF changes in Case 3. **(E)** CBF changes in Case 4. ASL, arterial spin labeling; CBF, cerebral blood flow; HDT, head down tilt.

To explore the differences in CBF changes between head down tilt position and lying flat position, 3 anterior circulation ischemic stroke patients (Case 1, 3 and 4) underwent arterial spin labeling examination. The CBF value under lying flat and head down tilt positions were subtracted and ranked. In most regions of Case 1 and Case 3, the CBF value under head down tilt position increased significantly compared to that under lying flat position (Figure 2). The CBF value in Case 4 also increased although not significant. The top 20 brain regions changed the most in each subject were shown in Figures 2C–E.

## Discussion

In AIS patients, various interventions were performed to improve CBF through collateral arteries, leptomeningeal recruitment, and increasing residual blood flow, aiming to improve cerebral perfusion and decrease injury within the ischemic penumbra region. But none so far has been demonstrated effective except reperfusion strategy. HeadPoST study demonstrated no difference in the functional recovery of patients assigned to flatting or sitting up position (6), which may be due to the broad inclusion of the patients (14). Head positioning trials should be performed in discrete patient cohorts with endpoints supported by pilot data (15). The present study first showed that the head down tilt position improved neurological function in acute ischemic stroke patients by possibly increasing the CBF of affected side.

We found that head down tilt treatment could prevent the further deterioration of clinical symptoms in AIS patients with END, because symptoms in all patients stop progression after the treatment. Meanwhile, improvements in NIHSS scoring were detected after head down tilt treatment and an increase in CBF of the ischemic area was induced in patients with anterior circulation infarction by head down tilt position. As we know, ischemic stroke is a potentially reversible process that is dependent on restoration of CBF within a time window of cellular viability that varies according to the severity and duration of the flow deficit. The prognosis of acute ischemic stroke was determined according to the location and size of the occluded cerebral vessel (1), the extent of collateral blood flow (1, 2) and the time to reperfusion therapy (22). Previous studies indicated that changes in head position could influence the prognosis of AIS patients through regulating cerebral perfusion pressure, intracranial pressure, cortical oxygenation (23), systolic and diastolic blood pressure, residual CBF to the ischemic brain tissue (24), collateral blood flow (25, 26), and mean velocity of the residual arterial blood flow (7). A study on “collateral therapeutics” in a rat stroke model of transient MCAO evaluated the effect of different strategies including induced hypertension, intravascular volume load, cerebral arteriolar vasodilation, and head down tilt on stroke outcome, and indicated that treatment with collateral therapeutics was associated with lower infarct volumes and higher chance of good functional outcome. Notably, the highest efficacy and safety profile was observed for head down tilt treatment (27). In addition, cerebral autoregulation ability was impaired in stroke patients and could lead to a decrease in CBF velocity during orthostatic stress with head-up tilt. Therefore, we argue that positioning AIS patients with head down tilt may exert neuroprotective effect through increasing CBF due to collateral circulation improvement by gravitational force (28). Furthermore, we argue that this benefit may be more obvious in the moderate stroke patients with LVO in the acute phase (within 24 h). For acute mild stroke, intensive antithrombosis should be best strategy to prevent the deterioration or reoccurrence of stroke (29). For severe ischemic stroke patients, recanalization as soon as possible may be the best way to acquire good outcome due to limited ischemic penumbra that can be salvaged. In addition, head down tilt treatment may further increase intracranial pressure for severe stroke. In the present study, 2 of the 4 patients had a posterior circulation stroke, which is expected to have a smaller response to collateral therapeutics due to less developed collaterals, compared to anterior circulation stroke. Taken together, we argue that the best target population should be acute moderate stroke patients with LVO, which is being determined by our ongoing trial (NCT03744533).

In previous studies, transcranial laser Doppler-recorded mean flow velocity (MFV) was usually used to reflect the changes in CBF after head positioning treatment. However, Transcranial Doppler only provided indirect information of CBF, due to measuring the flow velocity and not the volume flow. Therefore, changes of blood flow velocity could correspond to the change of CBF only if the vessel diameter was constant, which cannot be assumed a priori. Moreover, even very small changes in the position of the transcranial Doppler probe could alter the blood flow velocity readings. Furthermore, the relevance of changes in MFV to any improvement in clinical outcomes after AIS is uncertain (30). In our study, CBF was evaluated by arterial spin labeling, which steadily reflect the volume flow.

It was reported that serious adverse events of head down tilt treatment included visual field loss, cognitive aberrations, potentially life-threatening complications of the respiratory and cardiovascular systems (31). In our study, no serious adverse events, including aspiration pneumonia, were observed in patients treated with head down tilt position, which may be due to short-term and moderate tilt of head down position. Another important concern was about its effect on CBF, because decreased CBF may occur due to increased intracranial pressure (ICP) induced by head down tilt. The mechanism appeared to be related to the changes in venous outflow through the valueless jugular veins and vertebral venous plexuses. Cerebral venous and jugular venous pressures increase with head position lowering, leading to an increase in the cerebral venous blood volume and a subsequent increase in ICP. The cerebral perfusion pressure (CPP) was calculated as the difference between mean arterial pressure and ICP. The CPP may increase when head position was under the level of heart since the mean arterial pressure increased in this head down tilt position. We argued that in certain angle and duration time, head down tilt position exerted neuroprotective effect through increasing CPP. However, ICP increased due to the impairment of venous outflow in a greater minus angle, leading to the decrease in CPP and the deterioration of neurological function. The main limitation of the clinical study is the small sample, no control design.

## Conclusion

For the first time, the case series study suggests that head down tilt may exert neuroprotective effect in AIS patients possibly through increasing the cerebral perfusion. Due to its low cost and easy operation, the efficacy and safety of head down tilt position in acute ischemic patients are urgently needed to be confirmed in further clinical trials.

## Data availability statement

The raw data supporting the conclusions of this article will be made available by the authors, without undue reservation.

## Ethics statement

The studies involving human participants were reviewed and approved by the former General Hospital of Shenyang Military Region (No. k2017-10). The patients/participants provided their written informed consent to participate in this study.

## Author contributions

Z-AZ, N-NZ, and LT prospectively enrolled patients and acquired the data. ML, YC, and Z-AZ did statistical analysis. Z-AZ, N-NZ, and H-SC wrote the draft. S-LQ critically revised the manuscript. H-SC designed the study and critically edited the manuscript.

## Funding

This work was supported by grants from National Key R&D Program of China (2017YFC1308200) and the Science and Technology Project Plan of Liaoning Province (2018225023 and 2019JH2/10300027).

## Conflict of interest

The authors declare that the research was conducted in the absence of any commercial or financial relationships that could be construed as a potential conflict of interest.

## Publisher's note

All claims expressed in this article are solely those of the authors and do not necessarily represent those of their affiliated organizations, or those of the publisher, the editors and the reviewers. Any product that may be evaluated in this article, or claim that may be made by its manufacturer, is not guaranteed or endorsed by the publisher.
