# Supplementary material for: Effect of head-down tilt on clinical outcome and cerebral perfusion in ischemic stroke patients: A case series
Source: Front Neurol. 2022 Sep 26;13:992885. doi: 10.3389/fneur.2022.992885 (PMC9548884; doi:10.3389/fneur.2022.992885)
Supplement: Supplementary file 1 [file Image_1.PDF]

CARE case report guidelines

CARE Checklist of information to include when writing a case report

(cc) BY-NC-ND

Topic	Item	Checklist item description	Reported on Line
Title	1	The diagnosis or intervention of primary focus followed by the words "case report"	page1 Title
Key Words	2	2 to 5 key words that identify diagnoses or interventions in this case report, including "case report"	page 1 line 17,18
Abstract (no references)	3a	Introduction: What is unique about this case and what does it add to the scientific literature?	page 2 line 19-21
	3b	Main symptoms and/or important clinical findings	page 2 line 26-28
	3c	The main diagnoses, therapeutic interventions, and outcomes	page 2 line 22-25
	3d	Conclusion—What is the main "take-away" lesson(s) from this case?	page 2 line 29-31
Introduction	4	One or two paragraphs summarizing why this case is unique (**may include references**)	page3,4;line41-66
Patient Information	5a	De-identified patient specific information.	page3,4;line70-84
	5b	Primary concerns and symptoms of the patient.	Table 1
	5c	Medical, family, and psycho-social history including relevant genetic information	Table 1
	5d	Relevant past interventions with outcomes	Table 1
Clinical Findings	6	Describe significant physical examination (PE) and important clinical findings.	page6,7;line118-136
Timeline	7	Historical and current information from this episode of care organized as a timeline	page6,7;line118-136
Diagnostic Assessment	8a	Diagnostic testing (such as PE, laboratory testing, imaging, surveys).	page6,7;line118-136
	8b	Diagnostic challenges (such as access to testing, financial, or cultural)	page6,7;line118-136
	8c	Diagnosis (including other diagnoses considered)	page6,7;line118-136
	8d	Prognosis (such as staging in oncology) where applicable	page6,7;line118-136
Therapeutic Intervention	9a	Types of therapeutic intervention (such as pharmacologic, surgical, preventive, self-care)	page5;line86-92
	9b	Administration of therapeutic intervention (such as dosage, strength, duration)	page5;line86-92
	9c	Changes in therapeutic intervention (with rationale)	page5;line86-92
Follow-up and Outcomes	10a	Clinician and patient-assessed outcomes (if available)	page6,7;line118-136
	10b	Important follow-up diagnostic and other test results	page6,7;line118-136
	10c	Intervention adherence and tolerability (How was this assessed?)	page6;line119-128
	10d	Adverse and unanticipated events	page6;line119-128
Discussion	11a	A scientific discussion of the strengths AND limitations associated with this case report	page7-9;line139-203
	11b	Discussion of the relevant medical literature **with references**.	page7-9;line139-203
	11c	The scientific rationale for any conclusions (including assessment of possible causes)	page7-9;line139-203
	11d	The primary "take-away" lessons of this case report (without references) in a one paragraph conclusion	page10;line206-209
Patient Perspective	12	The patient should share their perspective in one to two paragraphs on the treatment(s) they received	page4;line70-74
Informed Consent	13	Did the patient give informed consent? Please provide if requested	Yes ☑ No ☐
